# Is there an over-prescription of psychotropic drugs to patients with Idiopathic Normal Pressure Hydrocephalus?

**DOI:** 10.1186/2045-8118-12-S1-O42

**Published:** 2015-09-18

**Authors:** Hanna Israelsson, Per Allard, Anders Eklund, Jan Malm

**Affiliations:** 1 Department of Pharmacology and Clinical Neuroscience, Umeå University, Umeå, Sweden; 2 Department of Clinical Science, division of Psychiatry, Umeå University, Umeå, Sweden; 3 Department of Radiation Sciences, Umeå University, Umeå, Sweden

## Introduction

Over-prescription of psychotropic drugs among elderly and cognitively impaired individuals is a well-known problem. One common side effect to a majority of these drugs is an increased risk to fall. This may be fatal in a population already susceptible for falling, such as INPH patients. The objective of this study was to describe the prevalence of prescribed antidepressants, antipsychotics, anxiolytics, hypnotics, and sedatives in shunted INPH patients compared with the population.

## Method

INPH patients consecutively shunted 2008-2010 in Sweden were scrutinized in one to three years after shunt surgery (in 2011) and patients remaining after inclusion (within 60-85 years and not having dementia, i.e., mini mental state examination≥23) had a standardized visit to their health-care giver and answered a questionnaire. Age- and sex-matched population-based controls underwent the same procedure. The prescription of psychotropic drugs and psychiatric diagnoses were obtained from the registers of the Swedish National board of Health and Welfare, where all diagnoses and prescribed medication are registered for all individuals receiving medical care in Sweden.

## Results

The study population consisted of 176 INPH patients and 368 controls. More INPH patients than controls received antidepressants (30% vs 11%, p<0.001). However, there was no difference between INPH patients and controls regarding a verified diagnosis of depression (2% vs 1%, p=0.40). More INPH patients than controls received anxiolytics, hypnotics, and sedatives (34% vs 25%, p=0.027). There was no difference between INPH patients and controls regarding antipsychotics (2% vs 1%).

## Conclusion

INPH patients received antidepressants, anxiolytics, hypnotics and sedatives in a higher degree than the general population despite the lack of a definitive psychiatric diagnosis. Considering the possible serious adverse effects of psychotropic drugs in cognitively impaired elderly, it is important to ensure that the prescribed medications are both adequate and safe when releasing an INPH patient from the hospital after shunting.

